# Supplementary material for: Giant topological magneto-optical effect in noncoplanar antiferromagnet
Source: Nat Commun. 2026 May 27;17:4409. doi: 10.1038/s41467-026-72889-5 (PMC13216597; doi:10.1038/s41467-026-72889-5)
Supplement: Supplementary file 1 — Supplementary Information [file 41467_2026_72889_MOESM1_ESM.pdf]

Supplementary Information for

"Giant topological magneto-optical effect in noncoplanar antiferromagnet"

Y. Okamura[1,2*†], Y. Hayashi[1†], N. D. Khanh[1],

Y. Tokura[1,3,4], S. Seki[1,2], and Y. Takahashi[1*]

[1]*Department of Applied Physics and Quantum Phase Electronics Centre, University of Tokyo, Tokyo 113-8656, Japan*

[2]*Research Centre for Advanced Science and Technology, University of Tokyo, Tokyo 153-8904, Japan*

[3]*RIKEN Centre for Emergent Matter Science (CEMS), Wako 351-0198, Japan*

[4]*Tokyo College, University of Tokyo, Tokyo 113-8656, Japan*

[*]To whom correspondence should be addressed (okamura@ap.t.u-tokyo.ac.jp, youtarou-takahashi@ap.t.u-tokyo.ac.jp)

[†]These authors equally contribute to this work.

Supplementary Note 1: Details of numerical Kramers–Kronig (KK) analysis

We summarize the workflow of numerical KK analysis in Supplementary Fig. 4a. The uncertainty in the KK analysis originates from how well the experimentally measured Kerr spectrum above 55 meV, $\theta_{\mathrm{K}}^{\mathrm{exp}}$, is reproduced by the Kerr spectrum calculated from an assumed Hall conductivity, $\theta_{\mathrm{K}}^{\mathrm{calc}}$. This agreement is quantified by the deviation Δ^2, where Δ is experimental noise level around 100 meV and set to be 0.65 mrad (Supplementary Fig. 4a). To be more specific, we illustrate three hypothetical $\sigma_{xy}(\omega)$ spectra in the energy range 0 – 85 meV, shown as red, blue, and green curves in Supplementary Fig. 4b. From each assumed $\sigma_{xy}(\omega)$, the corresponding $\theta_{\mathrm{K}}^{\mathrm{calc}}$ spectrum above 55 meV is obtained, as shown in Supplementary Fig. 4c. From these candidates, we can judge the consistency of the $\theta_{\mathrm{K}}^{\mathrm{calc}}$ spectra with $\theta_{\mathrm{K}}^{\mathrm{exp}}$ within experimental noise level; in this case, the green curve satisfies the criterion and is therefore adopted.

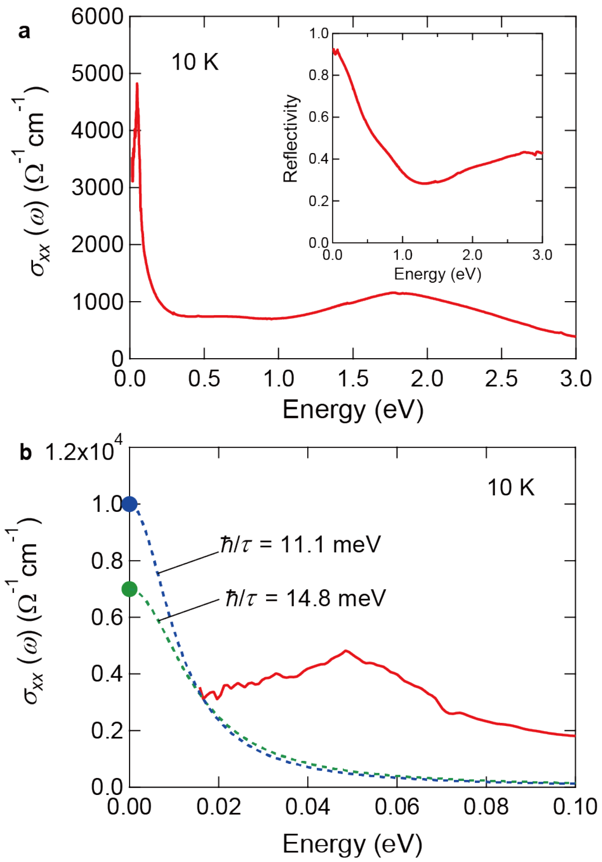

Supplementary Figure 1| Optical conductivity spectra. a Optical conductivity $\sigma_{xx}(\omega)$ spectrum, obtained from the reflectivity spectrum by Kramers-Kronig analysis down to 20 meV. (inset) Reflectivity spectrum at 10 K. **b** Magnified view of $\sigma_{xx}(\omega)$ below 0.1 eV (red curve). The blue and green circles at zero energy represent the typical d.c. conductivities $\sigma_{xx}(\omega = 0)$ from the transport measurements; the samples show a conductivity spread between 7000 and 10000 $\Omega^{-1}\text{cm}^{-1}$. We also show the simulated $\sigma_{xx}(\omega)$ spectra using the Drude model (blue and green dotted curves), for the two d.c. conductivity values. The scattering rate τ was chosen to connect the $\sigma_{xx}(\omega = 0)$ obtained from the transport measurement and $\sigma_{xx}(\omega)$ at the lowest measured photon energy (16 meV) from the optical measurement. On this basis, we extracted the upper bound of scattering rate $\hbar/\tau \sim 15$ meV; $\hbar$ is the reduced Planck constant.

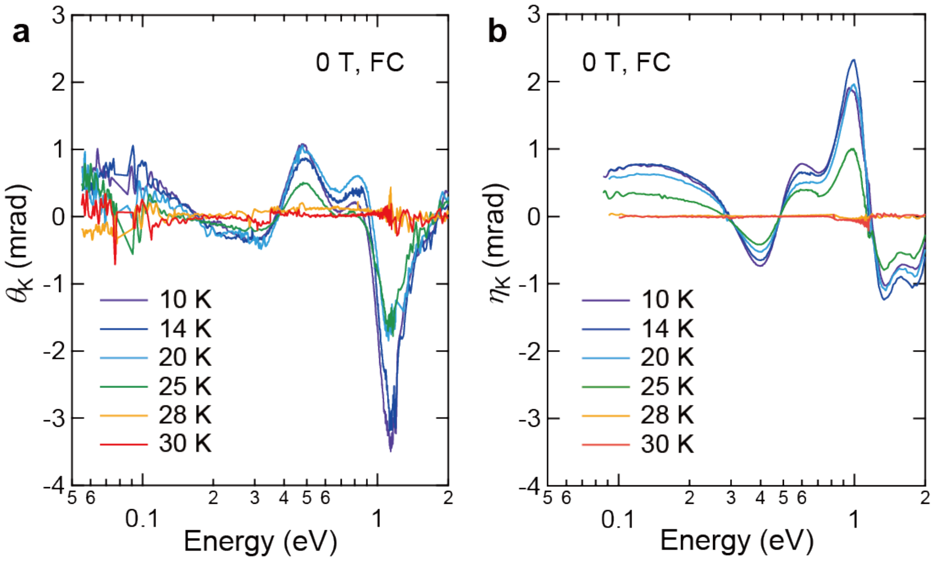

Supplementary Figure 2| MOKE spectra from far-infrared to visible region. a Kerr rotation $\theta_K(\omega)$ spectra from 55 meV to 2 eV. **b** Kerr ellipticity $\eta_K(\omega)$ spectra from 85 meV to 2 eV.

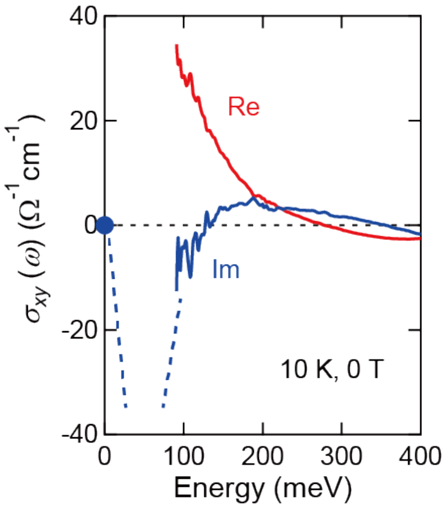

Supplementary Figure 3| Magnified view of low-energy $\sigma_{xy}(\omega)$ spectra deduced analytically. Real (red curve) and imaginary (blue curve) parts of optical Hall conductivity $\sigma_{xy}(\omega)$ at 10 K, 0 T. When decreasing the energy below 200 meV, Im $\sigma_{xy}(\omega)$ decreases with sign change from plus to minus value at ~ 120 meV, and Im $\sigma_{xy}(\omega = 0)$ must be zero as indicated by the blue circle because of causality constraint, which indicates the emergence of negative peak below 85 meV, as indicated by blue dotted lines.

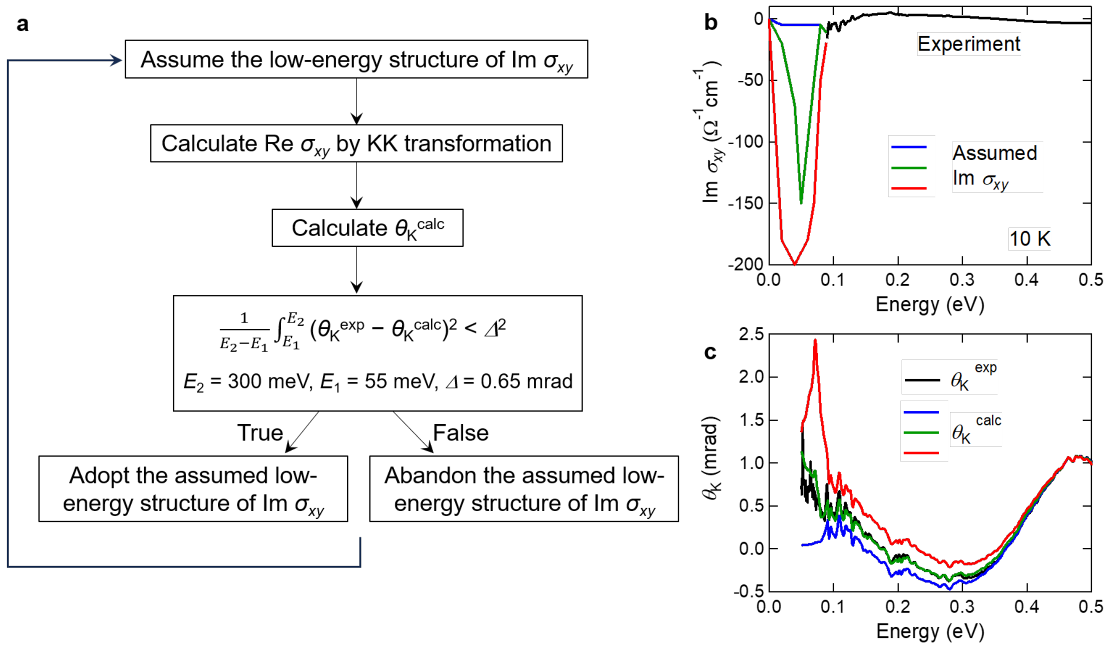

Supplementary Figure 4| Numerical Kramers Kronig analysis. **a** Workflow of KK analysis. **b** Some examples of assumed Im $\sigma_{xy}(\omega)$. **c** The experimentally measured $\theta_{\mathrm{K}}^{\mathrm{exp}}$ (black) and the $\theta_{\mathrm{K}}^{\mathrm{calc}}$ spectra calculated from assumed $\sigma_{xy}(\omega)$ (red, green and blue).

	Spin structure	Spectral region	Topological MOKE	Magnetization-induced MOKE	Magnetic field range
**$CoNb_3S_6$**	All-in all-out	Entire measured energy range (up to 2 eV)	~ 3.5 mrad	Too small to be resolved	Entire measured field range (0 – at least 3 T)
**Gd_2PdSi_3**	Skyrmion	Only low energy (up to 0.8 eV)	~ 0.3 mrad	Comparable to topological MOKE	Narrow range (~ 0.5 - 1 T)

Supplementary Table 1| Comparison of topological MOKE in $CoNb_3S_6$ and Gd_2PdSi_3. We summarize the characteristics of the topological MOKE in two representative materials, $CoNb_3S_6$ and Gd_2PdSi_3. Because of the intense Berry flux associated with the short-wavelength noncoplanar spin texture, the topological MOKE in $CoNb_3S_6$ is significantly more pronounced than that in Gd_2PdSi_3 in four key aspects: the spectral region where the topological MOKE is observed, the magnitude of the topological MOKE, its relative strength compared with the magnetization-induced MOKE, and the magnetic-field range over which it appears.
